# Neoadjuvant inetetamab and pertuzumab with taxanes and carboplatin (TCbIP) In locally advanced HER2-positive breast cancer: a prospective cohort study with propensity-matched analysis

**DOI:** 10.1186/s12885-024-12654-3

**Published:** 2024-07-22

**Authors:** Mingxia Jiang, Yue Chai, Jiaxuan Liu, Maiyue He, Yipeng Wang, Xue Yang, Zeyu Xing, Mengqi Zhang, Shihan Zhou, Fei Ma, Jiayu Wang, Peng Yuan, Binghe Xu, Qiao Li

**Affiliations:** 1 Department of Medical Oncology, National Cancer Center/National Clinical Research Center for Cancer/Cancer Hospital, Chinese Academy of Medical Sciences and Peking Union Medical College, Beijing, China; 2 Department of Breast Surgical Oncology, National Cancer Center/National Clinical Research Center for Cancer/Cancer Hospital, Chinese Academy of Medical Sciences and Peking Union Medical College, Beijing, China; 3 Department of VIP Medical Services, National Cancer Center/National Clinical Research Center for Cancer/Cancer Hospital, Chinese Academy of Medical Sciences and Peking Union Medical College, Beijing, China

**Keywords:** Inetetamab, Trastuzumab, HER2-positive breast cancer, Neoadjuvant, pCR

## Abstract

**Background:**

Inetetamab is the first domestically developed innovative anti-HER2 monoclonal antibody in China, proven effective and safe in HER2-positive advanced breast cancer. However, its efficacy and safety in neoadjuvant treatment of HER2-positive locally advanced breast cancer (LABC) remain to be validated.

**Methods:**

This prospective cohort study aimed to evaluate the efficacy and safety of inetetamab combined with pertuzumab, taxanes, and carboplatin (TCbIP) in neoadjuvant therapy for HER2-positive LABC, comparing it to data from patients treated with the TCbHP regimen (trastuzumab combined with pertuzumab, taxanes, and carboplatin) using propensity score matching (PSM). The primary endpoint was total pathological complete response (tpCR). Adverse events (AEs), objective response rate (ORR), and near-pCR were key secondary endpoints.

**Results:**

Forty-four patients with clinical stage IIA-IIIC HER2-positive LABC were prospectively enrolled and treated with the TCbIP regimen. The tpCR rate among 28 patients who completed surgery was 60.7%, comparable to and slightly higher than the TCbHP group in PSM (60.7% vs. 53.6%, *P* = 0.510). The ORR was 96.4%, and the DCR reached 100.0%. The most common ≥ grade 3 AE was neutropenia (21.4% vs. 11.9%, *P* = 0.350). No significant reduction in left ventricular ejection fraction was observed, and no patient withdrew from treatment due to AEs.

**Conclusion:**

Neoadjuvant therapy with TCbIP showed good efficacy and safety in patients with HER2-positive LABC and might be another promising option for neoadjuvant treatment.

**Trial registration:**

NCT05749016 (registration date: Nov 01, 2021).

**Supplementary Information:**

The online version contains supplementary material available at 10.1186/s12885-024-12654-3.

## Introduction

Human epidermal growth factor receptor 2 (HER2)-positive breast cancer is a subtype of breast cancer characterized by high invasiveness, a high risk of recurrence, and poor prognosis [1]. Due to gene amplification or protein overexpression of HER2, targeted therapy is the core treatment for HER2-positive breast cancer. The continuous development and successful marketing of anti-HER2 therapeutics [such as monoclonal antibodies, tyrosine kinase inhibitors (TKIs), and antibody–drug conjugates (ADCs)] have achieved good clinical efficacy in treating HER2-positive breast cancer and have gradually changed clinical practice [2, 3].

Neoadjuvant therapy for breast cancer is an essential part of the current comprehensive treatment of breast cancer, and it should be combined with molecular classification and clinical staging to select the appropriate patient population. Patients with locally advanced and inoperable conditions, those who are operable but do not meet the requirements of breast preservation or axillary lymph nodes preservation, and those who strongly desire the conservation of breast and axillary lymph nodes belong to the mandatory population of neoadjuvant therapy [4]. Additionally, a subset of the neoadjuvant population [e.g., patients with HER2-positive or triple-negative breast cancer (TNBC) with high tumor burden] can be used to guide postoperative adjuvant therapy based on the efficacy of neoadjuvant therapy [5]. With the gradual relaxation of indications for neoadjuvant patients, the successive publication of clinical research results, and the continuous update of treatment concepts, many viewpoints believe that all HER2-positive locally advanced breast cancer (LABC) patients who meet the criteria for single-target therapy with trastuzumab can consider dual-targeted therapy with trastuzumab combined with pertuzumab during the neoadjuvant therapy stage [6].

The results of the NeoSphere trial showed that, compared with the regimen of trastuzumab plus docetaxel, the THP regimen (pertuzumab, trastuzumab, and docetaxel) significantly improved the breast pathologic complete response (bpCR) rate (45.8% vs. 29%, *P* = 0.0141) and the 5-year invasive disease-free survival (iDFS) rate [86% vs. 81%, Hazard Ratio (HR) = 0.69] [7, 8]. There were no remarkable inter-group differences in treatment side effects. Therefore, trastuzumab plus pertuzumab therapy can increase the benefit of patients compared with single-trastuzumab therapy. The KRISTINE study [9] further confirmed that, compared with trastuzumab emtansine (T-DM1) + pertuzumab, the trastuzumab, pertuzumab, docetaxel, and carboplatin-based (TCbHP) regimen in neoadjuvant therapy for HER2-positive breast cancer demonstrated superior efficacy (pCR: 55.7% vs. 44.4%) and safety. TRYPHAENA study [10] revealed that there were no significant efficacy [total pCR (tpCR) rates: 51.9% vs. 45.3%] differences between the two groups [TCbHP vs. FEC-THP (5-fluorouracil + epirubicin + cyclophosphamide- docetaxel + trastuzumab + pertuzumab)], but the TCbHP group had a better safety effect (such as neutropenia and other toxic side effects). Guidelines recommend the TCbHP regimen as the preferred treatment for HER2-positive LABC with neoadjuvant indications.

Multiple clinical studies [11, 12] and meta-analyses [13–15] have demonstrated that HER2-positive LABC patients with neoadjuvant treatment who achieved pCR had significantly extended overall survival (OS) and DFS. Therefore, in the neoadjuvant therapy of HER2-positive breast cancer, how to further optimize the treatment strategy based on the TCbHP/THP regimen is a hot topic of clinical discussion. Inetetamab is the first self-developed anti-HER2 monoclonal antibody in China [16, 17]. Due to improved modification of the Fc domain, specifically differences in the amino acids at positions 359 and 361 in the constant region (inetetamab: D359, L361; trastuzumab: E359, M361), inetetamab exhibits equivalent binding activity and comparable affinity for the HER2 antigen. It also shares identical key quality attributes, such as inhibitory activity against in vitro cancer cell proliferation, protein folding, thermal stability, etc. (https://tbcr.amegroups.com/article/view/61051/html). Inetetamab demonstrates antitumor activity that is equivalent to trastuzumab in preclinical models [17]. The HOPES study demonstrated that the mPFS was 11.1 months with an objective response rate (ORR) of 61.5% in the postoperative recurrent-metastases first-line subgroup treated with inetetamab in combination with chemotherapy. For the multiline treatment group, the mPFS was 9.2 months, with an ORR remaining high at 46.7% [18]. In June 2020, inetetamab was approved for marketing in China in combination with vinorelbine in patients with HER2-positive metastatic breast cancer (MBC) who have received one or more chemotherapy regimens [19]. However, there was no robust evidence evaluating the combination of inetetamab with pertuzumab with chemotherapy (taxanes + carboplatin) in the neoadjuvant setting.

This study intends to replace trastuzumab with inetetamab (TCbIP) based on the TCbHP regimen and prospectively include HER2-positive LABC patients to evaluate the efficacy and safety of neoadjuvant therapy. To further assess the feasibility of the TCbIP regimen, we analyzed the efficacy and safety of HER2-positive LABC patients who had previously received neoadjuvant therapy with the TCbHP regimen at our center by propensity score matching (PSM) to promote the normalization of neoadjuvant therapy for HER2-positive LABC.

## Materials and methods

### Study design

**Fig. 1:**
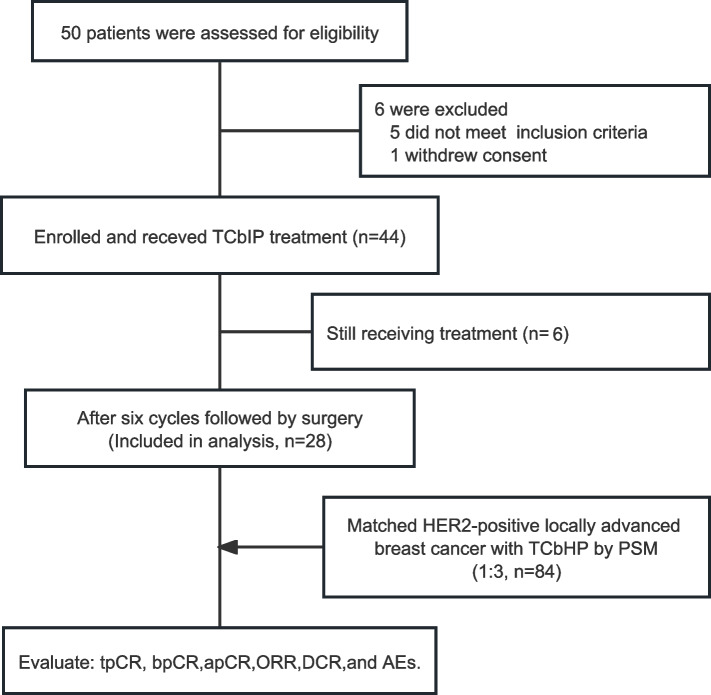
Trial flow diagram

This is a multicenter, prospective study initiated by the Cancer Hospital, Chinese Academy of Medical Sciences (CAMS) (ClinicalTrials.gov ID: NCT05749016). Clinical stage IIA-IIIC HER2-positive breast cancer patients, pathologically diagnosed by core biopsy at our center, were enrolled to receive neoadjuvant therapy of inetetamab and pertuzumab combined with taxanes plus carboplatin regimens (TCbIP), followed by surgery. Enrolled patients who underwent surgery were matched with stage IIA-IIIC HER2-positive patients who received neoadjuvant therapy of trastuzumab and pertuzumab combined with taxanes plus carboplatin regimens (TCbHP) at Cancer Hospital of CAMS during the same period (Fig. 1). Propensity score matching (PSM) was done with a ratio of 1:3 between the two therapy groups based on baseline T stage, N stage, and hormone receptor (HR) status characteristics.

The total pCR (tpCR, ypT0/Tis ypN0) was the primary endpoint. Adverse events (AEs), pCR in breast (ypT0/Tis) and axilla (ypN0) [bpCR: absence of invasive cancer cells in the breast tissue after surgery; apCR: absence of cancer cells in the axillary lymph nodes after surgery], objective response rate (ORR), near-pathological complete response (near-pCR, ypT1mi/1a/1bN0 or ypT0/isN1mi), and disease control rate (DCR) were secondary endpoints.

### Participants

Eligibility criteria included: (1) age between 18 and 75 year-old treatment-naïve women with newly diagnosed [clinical stage IIA-IIIC by the eighth edition of the American Joint Committee on Cancer (AJCC) of patients with breast cancer], invasive breast cancer with evaluable pathological lesions; (2) histologically confirmed HER2-positive [defined as 3 + staining intensity by immunohistochemistry (IHC) or HER2 IHC 2 + with fluorescence in situ hybridization (FISH) amplification] by accredited experts from the Pathology Department of Cancer Hospital, Chinese Academy of Medical Sciences and Peking Union Medical College, in accordance with the latest international guidelines and standards; (3) Eastern Cooperative Oncology Group (ECOG) performance status scored 0 or 1 and (4) adequate hematological, renal, hepatic and cardiac function.

Key exclusion criteria included: (1) stage IV (metastatic) disease; (2) previously received anti-tumor treatment; (3) Allergic to anti-HER2 target agents and/or its adjuvants, taxanes, carboplatin, etc.; (4) poorly controlled cardiovascular disease or a major cardiovascular event; (5) the presence of other active malignancies or severe concomitant disease and (6) female patients during pregnancy or lactation.

Informed consent was obtained from those who voluntarily chose to participate. For patients who did not meet the study criteria or chose not to participate, treatment was provided according to the current standard of care.

### Treatments

Patients in the TCbIP group received each 21‐day cycle of treatment consisting of taxanes (docetaxel 75 mg/m^2^, paclitaxel liposome 175 mg/m^2^, nab-paclitaxel 260 mg/m^2^) intravenously, carboplatin AUC = 4.5 intravenously, inetetamab 8 mg/kg loading dose followed by 6 mg/kg intravenously, and pertuzumab 840 mg loading dose followed by 420 mg intravenously for 6 cycles, followed by surgery. Treatment was discontinued if there was disease progression, unmanageable toxicity (assessed before each cycle), or if the physician or patient requested discontinuation. Matched patients in the TCbHP group received neoadjuvant therapy with trastuzumab 8 mg/kg loading dose, followed by 6 mg/kg intravenously (other drugs, dosages, and methods remained unchanged). Prophylaxis for hematologic toxicity with granulocyte colony-stimulating factor (G-CSF) was administered to patients in both groups.

Surgery was performed by surgical oncologists between two and six weeks after the completion of neoadjuvant therapy. Based on the pathological evaluation results (according to the AJCC eighth edition), the adjuvant treatment plan was formulated by specialist physicians. Follow-up was scheduled every three months for the first two years, then every six months during years three to five, and yearly thereafter.

### Assessment

The baseline clinical stage (based on the AJCC eighth) of the tumor was measured by physical palpation and imaging tests. Patients in both groups underwent palpation and imaging (ultrasound, CT, and MRI examinations) every two cycles (± seven days). Efficacy was evaluated according to Response Evaluation Criteria in Solid Tumors (RECIST) v1.1. ORR was defined as the percentage of complete response (CR) and partial response (PR). DCR was defined as the percentage of CR, PR, and SD. Patients with PD needed to withdraw from the study and the follow-up treatment strategies were adjusted according to the investigator's opinion.

Furthermore, tpCR was defined as no residual cancer cells or only ductal carcinoma in situ (DCIS) in breast specimens and no invasive cancer cells remaining in the regional lymph nodes. AEs were graded according to the National Cancer Institute—Common Terminology Criteria for Adverse Events (NCI—CTCAE) 5.0.

### Statistical analysis

The calculation of prospective phase II study population sample size (TCbIP): The primary endpoint of this phase II clinical trial is the pCR rate. Based on the pCR rate of THP regimen in the NeoSphere study [45.8% (95% CI 36.1–55.7)] and the PEONY study (in Aisa populations, 39.3%), the assumed pCR rate (historical control) is set at 40%. The maximum ineffective boundary value for the pCR rate is set at 40%, and the minimum effective boundary value for the pCR rate is set at 65%, with a power (1-β) of 80% and an α of 0.1 (one-sided). Based on these parameters, the calculated sample size is 17 cases. Considering potential dropouts, this prospective phase II study plans to enroll approximately 30 cases. If mid-term analysis reveals favorable safety and efficacy outcomes in the treatment group, along with promising trends, this phase II study permits an increase in sample size to further substantiate the stability and durability of the results.

This prospective cohort study using PSM initially included 28 HER2-positive LABC patients who received 6 cycles of neoadjuvant TCbIP therapy and subsequently underwent surgery (as of November 2023). PSM was done with the 1:3 nearest neighbor method using R version 4.2.1, for the TCbIP and TCbHP group respectively. The baseline stage T, stage N, and HR status characteristics were designed as the matching variables. Standardized mean differences (SMD) were used to assess the balance among the two treatment groups. The contingency of baseline characteristics between two matched treatment arms was evaluated, categorical variables were compared using the Pearson chi-square test or Fisher's exact test, and ranked variables were compared using the rank-sum test. The measurement data were represented as mean ± SD. The clinical efficacy, pathological efficacy, and AEs between two matched treatment arms were compared using the Pearson chi-square test or Fisher's exact test. Subgroup analyses were evaluated using Cochran's and Mantel–Haenszel's statistics, and included the following variables: age, HR status, HER2, tumor size, lymph node status, ki-67 expression level, histological grade, and clinical stage. GraphPad Prism version 9.4.0 was used to draw the forest diagram. Statistical analyses were carried out using IBM SPSS Statistics, version 28.0. All statistical tests were two-sided, and *P*-value < 0.05 was considered statistically significant.

## Results

### Patients and characteristics

Forty-four patients diagnosed with treatment-naïve HER2-positive breast cancer (clinical stage IIA-IIIC) were enrolled to receive neoadjuvant TCbIP therapy between November 2021 and November 2023. As of November 2023, a total of 28 patients received six cycles of neoadjuvant therapy and received sequential surgery. The median age at diagnosis in the TCbIP group was 53.0 years old (range, 30–72). According to the AJCC classification, 82.1% of patients were diagnosed at stage III. HR-positive patients accounted for 50% of the group. Eighty-four patients with HER2-positive breast cancer (clinical stage IIA-IIIC) who received the neoadjuvant TCbHP regimen during the same period at our center were matched using PSM. The two groups' SMD of matching covariates was improved and balanced (Table S1). The baseline characteristics of patients in the two matched treatment groups are listed in Table 1 (before PSM in Table S2).

**Table 1:** The baseline characteristics of patients between the two matched treatment groups

Characteristics	After propensity score matching			
	No. of patients (%)	TCbIP (*n* = 28, %)	TCbHP (*n* = 84, %)	*P* value
**Age at diagnosed, years**				0.148
Median age (range)		53 (30–72)	48 (26–72)	
≤ 35	10 (8.9)	2 (7.1)	8 (9.5)	
36–49	52 (46.4)	10 (35.7)	42 (50.0)	
≥ 50	50 (44.6)	16 (57.1)	34 (40.5)	
**Tumor size (cT)**				0.659
cT1c	8 (7.1)	1 (3.6)	7 (8.3)	
cT2	72 (64.3)	19 (67.9)	53 (63.1)	
cT3	20 (17.9)	4 (14.3)	16 (19.0)	
cT4	12 (10.7)	4 (14.3)	8 (9.5)	
**Lymph node status (cN)**				0.383
cN1	24 (21.4)	6 (21.4)	18 (21.4)	
cN2	45 (40.2)	14 (50.0)	31 (36.9)	
cN3	43 (38.4)	8 (28.6)	35 (41.7)	
**Stage (cTNM)**				1.000
II	20 (17.9)	5 (17.9)	15 (17.9)	
III	92 (82.1)	23 (82.1)	69 (82.1)	
**Ki-67**				0.202
< 50%	75 (67.0)	16 (57.1)	59 (70.2)	
≥ 50%	37 (33.0)	12 (42.9)	25 (29.8)	
**HR status**				1.000
HR-positive	56 (50.0)	14 (50)	42 (50.0)	
HR-negative	56 (50.0)	14 (50)	42 (50.0)	
**HER2**				0.350
IHC 2 + /FISH +	16 (14.3)	6 (21.4)	10 (11.9)	
IHC 3 +	96 (85.7)	22 (78.6)	74 (88.1)	
**Histological grade**				0.603
G2	47 (42.0)	17 (60.7)	30 (35.7)	
G3	41 (36.6)	7 (25.0)	34 (40.5)	
Unknown	24 (21.4)	4 (14.3)	20 (23.8)	

### Efficacy

The clinical response and pathological response evaluation after neoadjuvant chemotherapy (NAC) are presented in Table 2. The percentage of 28 enrolled patients with CR, PR, and SD after six therapeutic cycles was 14.3% (4 patients), 82.1% (23 patients), and 3.6% (1 patient), respectively. The ORR achieved 96.4% (27/28) and DCR achieved 100.0%. After PSM, the ORR was 94.0% (79/84) in the TCbHP group, while the DCR was 100.0%. The rate of modified radical mastectomy in the TCbIP group was comparable and slightly lower than in the TCbHP group (71.4% vs. 72.6%, *P* = 0.903). There was no statistical difference between the two groups.

**Table 2:** The clinical response after six therapeutic cycles and pathological response evaluations between matched TCbIP and TCbHP groups after PSM

Outcome	TCbIP, *n* = 28 (%)	TCbHP, *n* = 84 (%)	*P*
Clinical response evaluation			
CR	4/28 (14.3)	17/84 (20.2)	
PR	23/28 (82.1)	62/84 (73.8)	
SD	1/28 (3.6)	5/84 (6.0)	
PD	0/28 (0.0)	0/84 (0.0)	
ORR	27/28 (96.4)	79/84 (94.0)	1.000
DCR	28/28 (100.0)	84/84 (100.0)	1.000
Rate of modified radical mastectomy	20/28 (71.4)	61/84 (72.6)	0.903
Pathological response evaluation			
tpCR (ypT0/Tis ypN0)			0.510
pCR	17/28 (60.7)	45/84 (53.6)	
Non-pCR	11/28 (39.3)	39/84 (46.4)	
bpCR (ypT0/is)			0.506
pCR	18/28 (64.3)	48/84 (57.1)	
Non-pCR	10/28 (35.7)	36/84 (42.9)	
apCR (ypN0)			0.460
pCR	22/28 (78.6)	60/84 (71.4)	
Non-pCR	6/28 (21.4)	24/84 (28.6)	
near-pCR (ypT1mi/1a/1bN0 or ypT0/isN1mi)	4/28 (14.3)	11/84 (13.1)	1.000

**Fig. 2:**
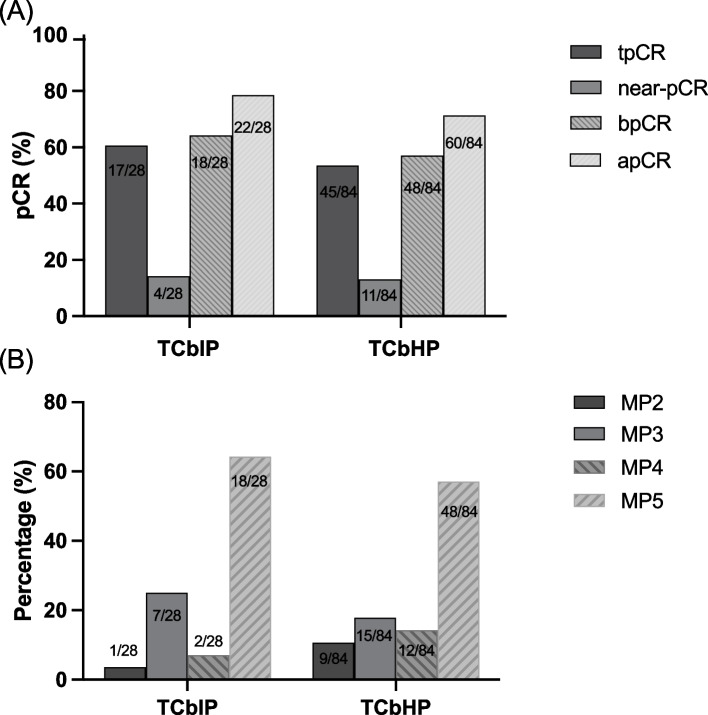
Pathological complete response. **A** pCR rate in TCbIP and TCbHP group. **B** The Miller–Payne (MP) grade associated with the pathological response evaluation of the two group patients

In recent years, many studies have confirmed that patients who achieve pCR or near-pCR (ypT1mi/1a/1bN0 or ypT0/isN1mi) after NAC in breast cancer predict a better prognosis [20]. In this study, the tpCR (ypT0/TisN0) and near-pCR rates in the TCbIP group reached 60.7% (17/28) and 14.3% (4/28). After PSM, there was no statistical difference between the two groups, but pathological efficacy was better in the TCbIP group (tpCR: 60.7% vs. 53.6%, *P* = 0.510; near-pCR: 14.3% vs. 13.1%). Further analysis of the bpCR and apCR rates were also slightly higher in the TCbIP group (64.3% vs. 57.1%, *P* = 0.506; 78.6% vs. 71.4%, *P* = 0.460; respectively) (Fig. 2A). Figure 2B shows the results of the Miller–Payne (MP) grade for factors associated with pathological response evaluation of the two group patients. Twenty-seven patients achieved ≥ 3 MP grade (96.4%).

### Clinical-pathological factors of pCR rate

**Fig. 3:**
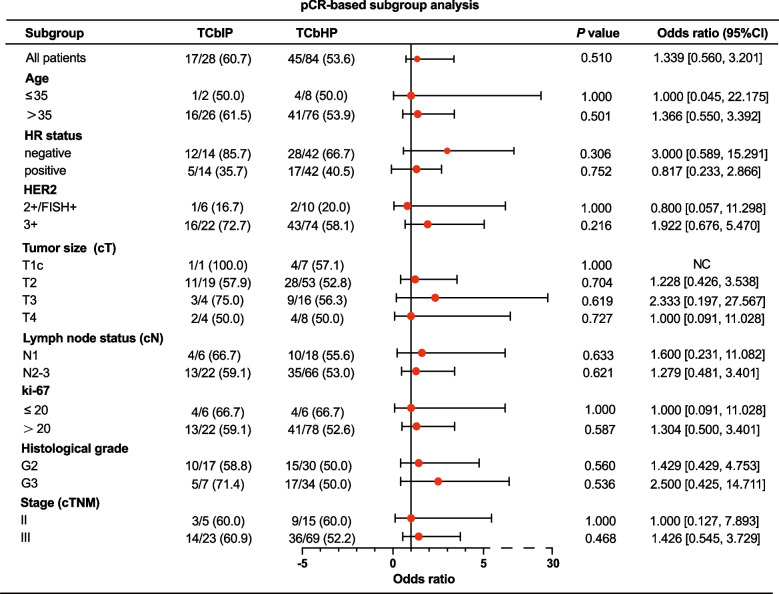
Subgroup analysis based on tpCR between matched TCbIP and TCbHP group

To investigate the impact of clinic-pathological factors on the pCR rate of neoadjuvant therapy for HER2-positive breast cancer, subgroup analyses showed that there was no statistically significant difference in the impact of multiple factors on the tpCR rate of patients (Fig. 3). However, HER2-positive breast cancer patients with HR-negative status, T3 (cT > 5 cm), or histological grade 3 might achieve a higher pCR rate [Odds ratio (OR) > 2] with the TCbIP regimen compared to TCbHP.

Meanwhile, stratified subgroup analysis based on bpCR (Fig. S1) and apCR (Fig. S2) showed that the TCbIP regimen seemed to be more likely to obtain bpCR in the HR-negative population [OR = 2.400, 95% confidence interval (CI): 1.466–12.370, *P* = 0.476; OR = 3.059, 95% CI: 0.348–26.921, *P* = 0.529].

Furthermore, the apCR rate of the population of HER2 IHC 3 + using the TCbIP regimen appeared to be superior. However, there was no statistically significant difference between them. Fig. S3 shows changes in HER2 and HR status after neoadjuvant dual-targeted therapy. The total population had 50 patients with HER2-positive residual disease (RD) after neoadjuvant therapy (TCbIP: 11 patients; TCbHP: 39 patients). Excluding patients with minimal RD insufficient for IHC, one patient (12.5%) of the TCbIP group had HER2 3 + RD, 5 (62.5%) had HER2 2 + /FISH + RD, and 2 (25.0%) had HER2 1 + RD (Fig. S3A). In terms of inconsistent HR status, there were 2 patients with inconsistent HR status. In the TCbIP treatment group, of the 6 patients with HR-positive residual tumors on core biopsies, one became negative after neoadjuvant therapy. Of the 2 patients with residual tumors who had HR-negative core biopsies, one became positive after neoadjuvant therapy. The TCbHP group showed a similar trend (Fig. S3B).

### Safety

Twenty-eight patients treated with TCbIP were evaluated for AEs. Treatment toxicities are shown in Table 3. The TCbIP regimen was mainly associated with grade 1–2 AEs (anemia, nausea, leukopenia, thrombocytopenia, etc.). The most common severe complications (grade 3 or 4) were neutropenia (6/28, 21.4%), all of which were considered related to chemotherapy drugs. In addition, no cardiac-related AEs occurred in any of the enrolled patients. Seven patients (25.0%) had their dose of chemotherapy drugs reduced. All AEs recovered after symptomatic treatment; no death or life-threatening events occurred, and the overall safety was controllable. The incidence of AEs was similar among matched patients, with no statistically significant differences between the two groups.

**Table 3:** Treatment toxicity between TCbIP and TCbHP group

Toxicity	Grade 1or 2			Grade 3 or 4		
	**TCbIP**	**TCbHP**	***P***	**TCbIP**	**TCbHP**	***P***
Anemia	24 (85.7)	62 (73.8)	0.196	0	3 (3.6)	0.735
Leukopenia	11 (39.3)	29 (34.5)	0.649	2 (7.1)	3 (3.6)	0.792
Neutropenia	8 (28.6)	22 (26.2)	0.805	6 (21.4)	10 (11.9)	0.350
Febrile neutropenia	-	-	-	1 (3.6)	2 (2.4)	1.000
Thrombocytopenia	11 (39.3)	18 (21.4)	0.062	0	3 (3.6)	0.735
ALT increased	7 (25.0)	31 (36.9)	0.249	0	2 (2.4)	1.000
AST increased	8 (28.6)	34 (40.5)	0.260	0	0	-
Creatinine increased	2 (7.1)	13 (15.5)	0.423	0	0	-
Diarrhea	7 (25.0)	20 (23.8)	0.899	0	0	-
Nausea	19 (67.9)	48 (57.1)	0.317	1 (3.6)	0	0.562
Vomiting	1 (3.6)	6 (7.1)	0.822	0	0	-
neuropathy peripheral	1 (3.6)	10 (11.9)	0.359	0	0	-
Abdominal pain upper	2 (7.1)	4 (4.8)	1.000	0	0	-
Mucositis	6 (21.4)	9 (10.7)	0.262	0	0	-
Cardiac dysfunction	0	0	-	0	0	-

*ALT* Alanine aminotransferase, *AST *Aspartate aminotransferase

Furthermore, nausea (48/84, 57.1%) was the most common nonhematological AE for patients who received TCbHP therapy. Hematologic toxicity such as neutropenia (10/84, 11.9%), anemia (3/84, 3.6%), leukopenia (3/84, 3.6%), and thrombocytopenia (3/84, 3.6%) were the most common grade ≥ 3 AEs. Similarly, no patients had a decrease in left ventricular ejection fraction of more than 10%. Regarding peripheral neuropathy, only one patient was observed in the TCbIP group, compared to 10 of 84 patients in the TCbHP group, possibly due to the use of nab-paclitaxel. Overall, compared to trastuzumab, the inetetamab-treated group had a higher incidence of nausea, anemia, thrombocytopenia, and mucositis (more than 10%). However, all levels of AEs were not comparable between the two groups. And most of the AEs that occurred in the TCbIP group were grade 1–2, which were usually acceptable.

## Discussion

Neoadjuvant therapy is an important component of the comprehensive management of early breast cancer. It not only makes surgery possible for inoperable patients but also reduces the extent of surgery, increases the likelihood of breast-conserving or axilla-conserving surgery, and lowers the rate of postoperative recurrence. In addition, the development of adjuvant treatment strategies guided by the efficacy of neoadjuvant therapy can contribute to the long-term survival of patients [21]. HER2 is a significant biological indicator affecting the prognosis of breast cancer patients [22]. With the advent of precision treatment of breast cancer based on molecular typing and the deepening of treatment concepts, the development of targeted drugs has progressively improved.

the survival benefit for HER2-positive breast cancer patients. Several clinical studies and meta-analyses have pointed out that pCR is an important efficacy predictor and prognostic indicator for patients with HER2-positive breast cancer [23, 24]. Therefore, optimizing treatment modalities and therapeutic strategies to achieve the best outcomes for patients is a major research focus in the neoadjuvant treatment field of HER2-positive breast cancer.

Both the TRYPHAENA [10] as well as the KRISTINE [9] studies demonstrated the efficacy (pCR: 51.9% and 55.7%) and safety of the TCbHP regimen (six cycles) in the neoadjuvant treatment of HER2-positive breast cancer. The TRAIN-2 study also revealed that in the context of dual-target therapy, anthracycline-free did not affect patient prognosis. The TCbHP regimen resulted in the same pCR rate compared to the anthracycline-containing regimen (5-fluorouracil + epirubicin + cyclophosphamide + HP * three cycles → paclitaxel + carboplatin + HP * 6 cycles, FEC * 3 → TCbHP * 6), but with a significantly lower toxic response such as neutropenia (10% vs. 1%, *p* < 0.0001) [25]. Therefore, the TCbHP regimen has now become the standard treatment choice for neoadjuvant treatment of HER2-positive breast cancer.

Inetetamab (Cipterbin®) is the first innovative anti-HER2 monoclonal antibody, independently developed by Sunshine Guojian Pharmaceutical (Shanghai) Co., Ltd. in China. It is not a biosimilar of the trastuzumab monoclonal antibody but has the same anti-HER2 effect as trastuzumab. A phase II prospective clinical study showed that the regimen (inetetamab + pyrotinib + vinorelbine) has a manageable safety profile with a mPFS of 8.63 months (95% CI: 4.15–13.12) in 30 patients with HER2-positive MBC who had disease progression after prior treatment with trastuzumab [26]. Researchers retrospectively also analyzed the efficacy and safety of inetetamab in HER2-positive advanced breast cancer (ABC) in real clinical practice [19, 27]. Thus, the combination treatment based on inetetamab for HER2-positive MBC has a favorable efficacy and safety profile and provides a viable alternative treatment option for patients with HER2-positive ABC.

In this prospective cohort study, we firstly investigated to evaluate the efficacy and safety of inetetamab + pertuzumab + taxanes + carboplatin (TCbIP) as a neoadjuvant chemotherapy regimen in the treatment of patients with locally advanced HER2-positive breast cancer. The ORR of 28 patients was 96.4% and the DCR was 100.0%. After matching HER2-positive LABC patients (*n* = 84) treated with neoadjuvant therapy with the TCbHP regimen applied in our center by propensity score, there was a 7.1% increase in tpCR (ypT0/Tis ypN0) in the TCbIP group (*P* = 0.510), a 7.2% increase in bpCR (*P* = 0.506), and a 7.2% increase in apCR (*P* = 0.460). The preliminary results of this study suggest that the TCbIP regimen has a superior elevation in pCR rates in HER2-positive LABC. Although the pCR rate did not reach statistical significance, it indicates that inetetamab exhibits favorable efficacy like trastuzumab in neoadjuvant treatment of HER2-positive LABC, which warrants further exploration in subsequent studies. Increasing the sample size in the future may demonstrate that inetetamab achieves non-inferior efficacy benefits (compared to trastuzumab).

Additionally, we utilized different types of taxanes (nab-paclitaxel, docetaxel, and paclitaxel liposome) in the study, but this is unlikely to affect the comparability of the TCbIP and TCbHP regimens. This is because patients included retrospectively from a single center who received TCbHP treatment also received various types of taxanes. Furthermore, despite these drugs having different formulations and pharmacokinetic properties, they have demonstrated similar efficacy in anticancer therapy. In clinical practice, we strictly adhere to recommended dose intensity based on the patient's body surface area. The use of multiple taxane formulations reflects real-world clinical practices and enhances the external validity of our study findings. This provides a certain theoretical basis for our comparisons.

Previous studies have found that common AEs associated with the “trastuzumab + pertuzumab” neoadjuvant regimen, including neutropenia, diarrhea, nausea, vomiting, anemia, and increased alanine aminotransferase (ALT) and aspartate aminotransferase (AST) levels [28]. In this study, the most common AEs in the TCbIP treatment group included anemia, nausea, leukopenia, and decreased neutrophil count. Notably, grade ≥ 3 neutropenia occurred in more than 20% of patients, and febrile neutropenia was observed in 3.6%. Hematologic toxicity was the primary reason for dose reduction. However, all AEs were manageable with symptomatic treatment, and no fatal or life-threatening events occurred. Overall, the TCbIP regimen did not expand the AE profile compared with trastuzumab.

Previous studies have indicated that the growth of HR-negative/HER2-positive breast cancer may be highly dependent on the HER2 gene [29], resulting in a high pCR rate after neoadjuvant anti-HER2 therapy. Natsuki T et al. [30] found that the percentage of ER-negative and HER2-positive tumor cells was independently associated with the pCR rate in ER-positive and HER2-positive breast cancer. In our subgroup analysis, HR-negative patients showed increased benefit after treatment with the TCbIP regimen compared to the TCbHP group, as evidenced by the analysis of influencing factors based on tpCR, bpCR, and apCR (OR > 2, *P* > 0.05). Inetetamab showed superior efficacy in patients with several high-risk factors, such as Ki-67 > 20%, histological grade III, and lymph node metastasis. Despite these promising results, statistical significance was not achieved, indicating the need for larger sample sizes in future exploratory research.

Anti-HER2 targeted therapies are crucial in treating HER2-positive breast cancer, including neoadjuvant therapy, adjuvant therapy, intensive adjuvant therapy for non-pCR patients, and salvage therapy for late-stage disease. The KATHERINE study [12] redefined the standard of care for adjuvant targeted therapy, showing that T-DM1 in the adjuvant phase of HER2-positive patients with non-pCR could achieve a 50% reduction in the risk of recurrence, with an absolute 3-year iDFS benefit of up to 11.3%. Furthermore, the ExteNET trial confirmed that extending the adjuvant regimen with the tyrosine kinase inhibitor (TKI) neratinib for one year increased the clinical benefit of anti-HER2 adjuvant therapy, particularly in HR-positive/HER2-positive breast cancer patients [31]. However, studies have shown that the presence of HER2-negative tumors in residual disease is associated with poorer prognosis than HER2-positive tumors remaining after neoadjuvant therapy [32, 33]. Loss of ER positivity was also an independent prognostic factor for poorer DFS and OS[34]. Therefore, HR and HER2 status should be retested for RD to inform further treatment decisions. In this study, a pooled analysis of HR and HER2 status was performed in patients who had postoperative non-pCR whose tumors were available for IHC. Postoperative changes in HR and HER2 status had similar outcomes in both populations, with 87.5% of the TCbIP group remaining HER2-positive and 75.0% remaining HR-positive.

It should be further emphasized that this study is a prospective study, and its results were compared with data from patients who received treatments outside the trial. The study found that the TCbIP regimen has good efficacy and safety in HER2-positive LABC patients and has certain advantages compared to the TCbHP regimen. However, since the data of this study were obtained through a prospective cohort study (a small sample size) and compared with historical data, further validation of these results is needed in larger-scale randomized controlled trials. Secondly, this is the limitation of this study, which only evaluated short-term efficacy. In the future, we still need to further identify the survival benefit by long-term survival follow-up.

In summary, while dual-target-based neoadjuvant therapy with trastuzumab and pertuzumab is current clinical practice, we must consider future options. Inetetamab may also be an option for neoadjuvant treatment of HER2-positive breast cancer patients, considering the combination of chemotherapeutic agents, the appropriate treatment duration, and the management of safety and adverse effects during the neoadjuvant phase.

## Conclusion

This study confirmed that the TCbIP regimen demonstrated non-inferior efficacy and a modestly improved pCR rate compared to the TCbHP regimen. Moreover, the TCbIP regimen did not broaden the spectrum of AEs in patients. Therefore, the TCbIP regimen represents a promising alternative strategy for the neoadjuvant treatment of HER2-positive LABC, warranting further investigation to expand treatment options for patients and inform clinical decision-making.

### Supplementary Information

Supplementary Material 1: Table S1. The standardized mean difference improvement between the two groups after propensity score matching.

Supplementary Material 2: Table S2. The baseline characteristics status of the two groups before PSM.

Supplementary Material 3: Figure S1. Subgroup analysis based on bpCR between matched TCbIP and TCbHP group.

Supplementary Material 4: Figure S2. Subgroup analysis based on apCR between matched TCbIP and TCbHP group.

Supplementary Material 5: Figure S3. Changes in HER2 expression and HR status between TCbIP and TCbHP group, following neoadjuvant treatment. (A) The Sankey diagrams show the changes in two groups with HER2 expression. (B) The Sankey diagrams show the changes in two groups with HR status.

## Data Availability

The data that support the findings of this study are available on request from the corresponding author.

## Abbreviations

ADC: Antibody–drug conjugate
ADCC: Antibody-dependent cell-mediated cytotoxic
AEs: Adverse events
AJCC: American Joint Committee on Cancer
AST/ALT: Alanine aminotransferase/ aspartate aminotransferase
bpCR: Breast pathologic complete response
CI: Confidence interval
CR: Complete response
DCIS: Ductal carcinoma in situ
DCR: Disease control rate
G-CSF: Granulocyte colony-stimulating factor
HER2: Human epidermal growth factor receptor 2
iDFS: Invasive disease-free survival
LABC: Locally advanced breast cancer
MBC: Metastatic breast cancer
mPFS: Median PFS
MP: Miller-Payne
NAC: Neoadjuvant chemotherapy
NCI-CTCAE: National Cancer Institute-Common Terminology Criteria for Adverse Events
near-pCR: Near-pathological complete response
ORR: Objective response rate
OS: Overall survival
PR: Partial response
PSM: Propensity score matching
RD: Residual disease
RECIST: Response Evaluation Criteria in Solid Tumors
SABCS: San Antonio Breast Cancer Symposium
SMD: Standardized mean differences
T-DM1: Trastuzumab emtansine
TKIs: Tyrosine kinase inhibitors
TNBC: Triple-negative breast cancer
tpCR: Total pCR
