# Supplementary material for: Neoadjuvant inetetamab and pertuzumab with taxanes and carboplatin (TCbIP) In locally advanced HER2-positive breast cancer: a prospective cohort study with propensity-matched analysis
Source: BMC Cancer. 2024 Jul 22;24:877. doi: 10.1186/s12885-024-12654-3 (PMC11265051; doi:10.1186/s12885-024-12654-3)
Supplement: Supplementary file 1 — Supplementary Material 1: Table S1. The standardized mean difference improvement between the two groups after propensity score matching. [file 12885_2024_12654_MOESM1_ESM.docx]

|  | Standardized mean difference | | |
| --- | --- | --- | --- |
| matching covariate | unadjusted | PSM | Percent balance improvement |
| HR | 0.0143 | 0 | 100.0% |
| cT | -0.0474 | 0 | 100.0% |
| cN | 0.3307 | 0 | 100.0% |

**Supplementary table S1.** The standardized mean difference improvement between the two groups after propensity score matching.

HR: estrogen receptor (ER) / progesterone receptor (PgR) status; cT: tumor size, cN: lymph node status.
