# Supplementary material for: Neoadjuvant inetetamab and pertuzumab with taxanes and carboplatin (TCbIP) In locally advanced HER2-positive breast cancer: a prospective cohort study with propensity-matched analysis
Source: BMC Cancer. 2024 Jul 22;24:877. doi: 10.1186/s12885-024-12654-3 (PMC11265051; doi:10.1186/s12885-024-12654-3)
Supplement: Supplementary file 2 — Supplementary Material 2: Table S2. The baseline characteristics status of the two groups before PSM. [file 12885_2024_12654_MOESM2_ESM.docx]

**Supplementary table S2.** The baseline characteristics status of the two groups before PSM.

| **Characteristics** | **Before propensity score matching** | | | |
| --- | --- | --- | --- | --- |
|  | **No. of patients**  **(%)** | **TCbIP (n=28, %)** | **TCbHP**  **(n=140, %)** | ***P* value** |
| **Age at diagnosed, years** |  |  |  | 0.154 |
| Median age (range) |  | 53 (30-72) | 48 (21-72) |  |
| ≤35 | 17 (10.1) | 2 (7.1) | 15 (10.7) |  |
| 36-49 | 76 (45.2) | 10 (35.7) | 66 (47.1) |  |
| ≥50 | 75 (44.6) | 16 (57.1) | 29 (20.7) |  |
| **Tumor sized (cT)** |  |  |  | 0.577 |
| cT1c | 17 (10.1) | 1 (3.6) | 16 (11.4) |  |
| cT2 | 100 (59.5) | 19 (67.9) | 81 (57.9) |  |
| cT3 | 37 (22.0) | 4 (14.3) | 33 (23.6) |  |
| cT4 | 14 (8.3) | 4 (14.3) | 10 (7.1) |  |
| **Lymph node status** |  |  |  | 0.645 |
| cN1 | 55 (32.7) | 6 (21.4) | 49 (35.0) |  |
| cN2 | 57 (33.9) | 14 (50.0) | 43 (30.7) |  |
| cN3 | 56 (33.3) | 8 (28.6) | 48 (34.3) |  |
| **Stage (cTNM)** |  |  |  | 0.243 |
| II | 45 (26.8) | 5 (17.9) | 40 (28.6) |  |
| III | 123 (73.2) | 23 (82.1) | 100 (71.4) |  |
| **Ki-67** |  |  |  | 0.521 |
| ＜50% | 105 (62.5) | 16 (57.1) | 89 (63.6) |  |
| ≥50% | 63 (37.5) | 12 (42.9) | 51 (36.4) |  |
| **HR** |  |  |  | 0.945 |
| HR-positive | 83 (49.4) | 14 (50) | 69 (49.3) |  |
| HR-negative | 85 (50.6) | 14 (50) | 71 (50.7) |  |
| **HER2** |  |  |  | 0.316 |
| IHC 2+/FISH + | 23 (13.7) | 6 (21.4) | 17 (12.1) |  |
| IHC 3+ | 145 (86.3) | 22 (78.6) | 123 (87.9) |  |
| **Histological grade** |  |  |  | 0.587 |
| G2 | 67 (39.9) | 17 (60.7) | 50 (35.7) |  |
| G3 | 53 (31.5) | 7 (25.0) | 46 (32.9) |  |
| unknown | 48 (28.6) | 4 (14.3) | 44 (31.4) |  |
