# Supplementary figures and images for: Neoadjuvant inetetamab and pertuzumab with taxanes and carboplatin (TCbIP) In locally advanced HER2-positive breast cancer: a prospective cohort study with propensity-matched analysis
Source: BMC Cancer. 2024 Jul 22;24:877. doi: 10.1186/s12885-024-12654-3 (PMC11265051; doi:10.1186/s12885-024-12654-3)

bpCR-based subgroup analysis

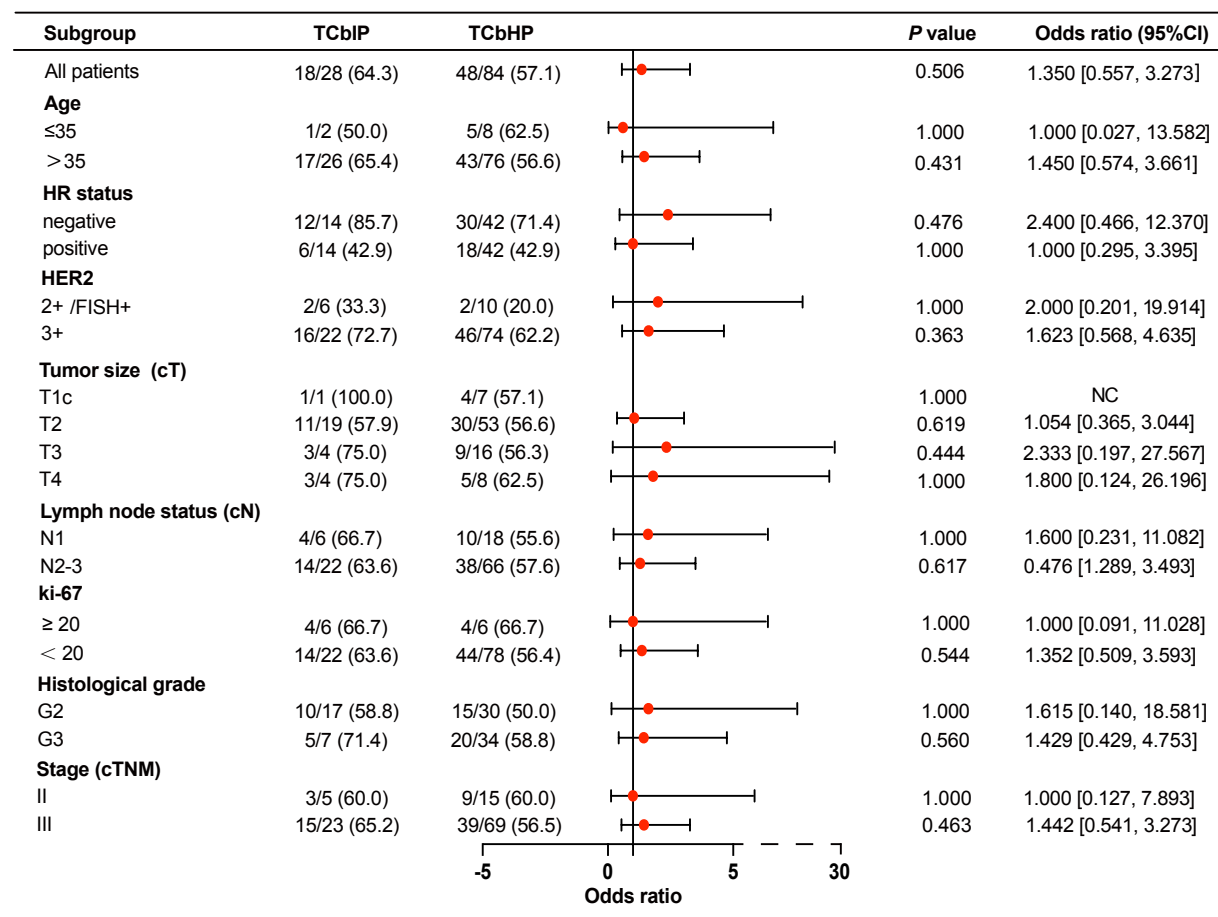

Supplement: Supplementary file 3 — Supplementary Material 3: Figure S1. Subgroup analysis based on bpCR between matched TCbIP and TCbHP group. [file 12885_2024_12654_MOESM3_ESM.pdf]

apCR-based subgroup analysis

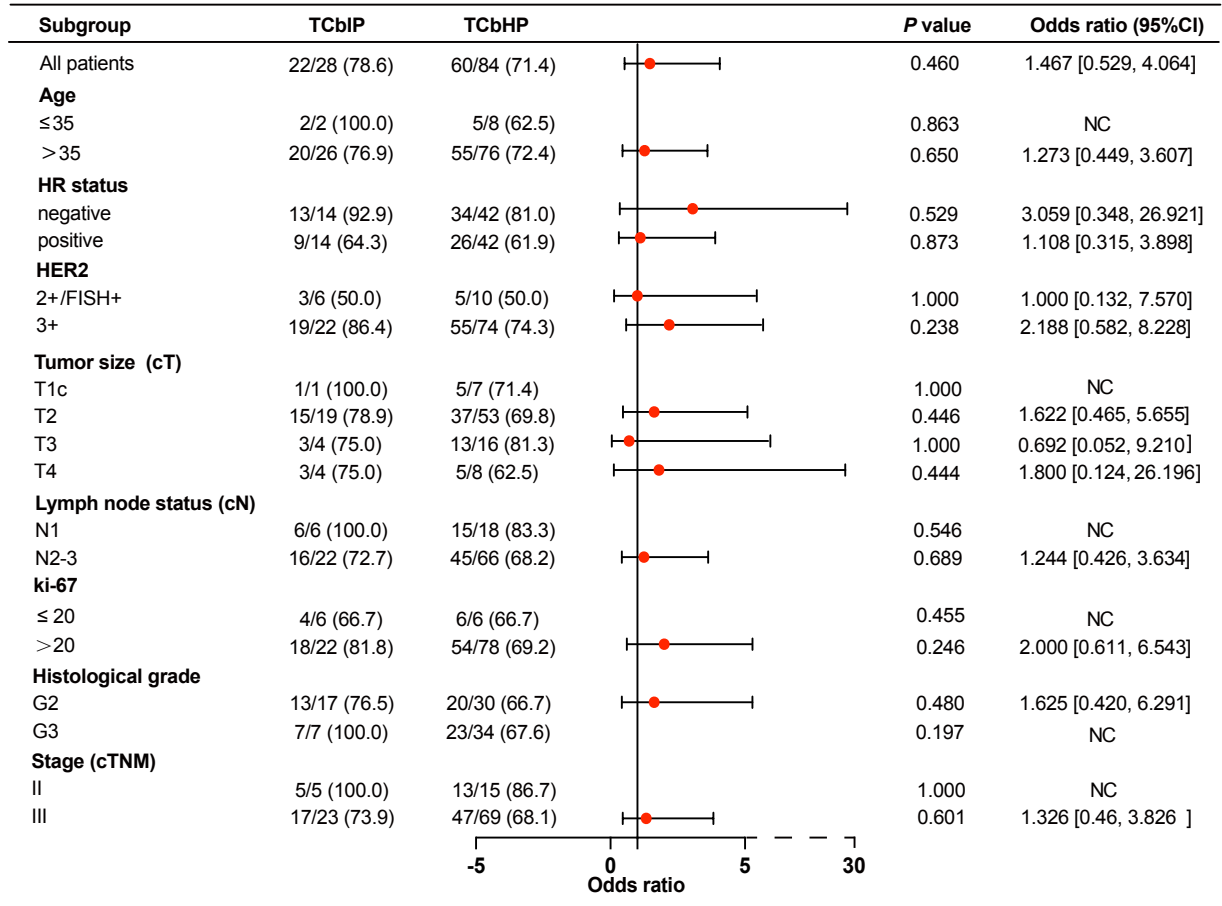

Supplement: Supplementary file 4 — Supplementary Material 4: Figure S2. Subgroup analysis based on apCR between matched TCbIP and TCbHP group. [file 12885_2024_12654_MOESM4_ESM.pdf]

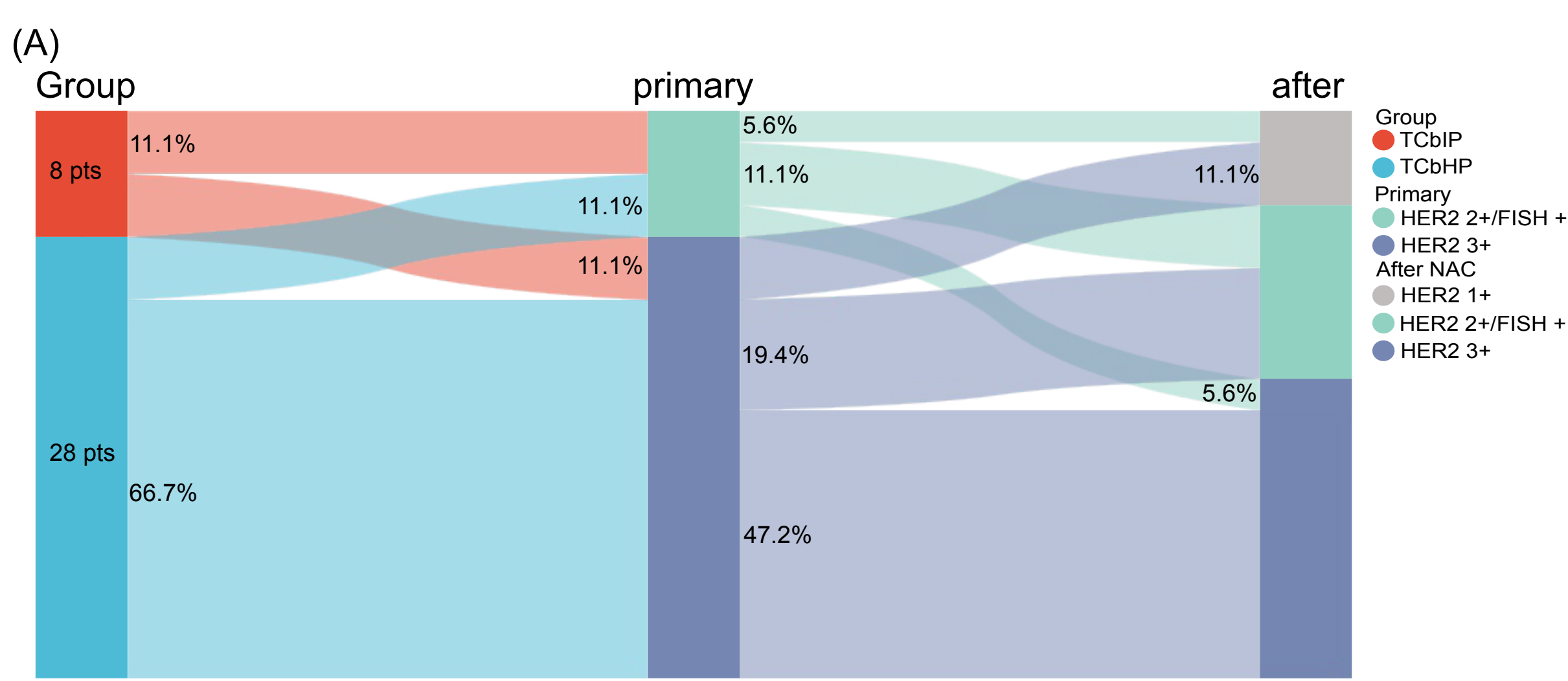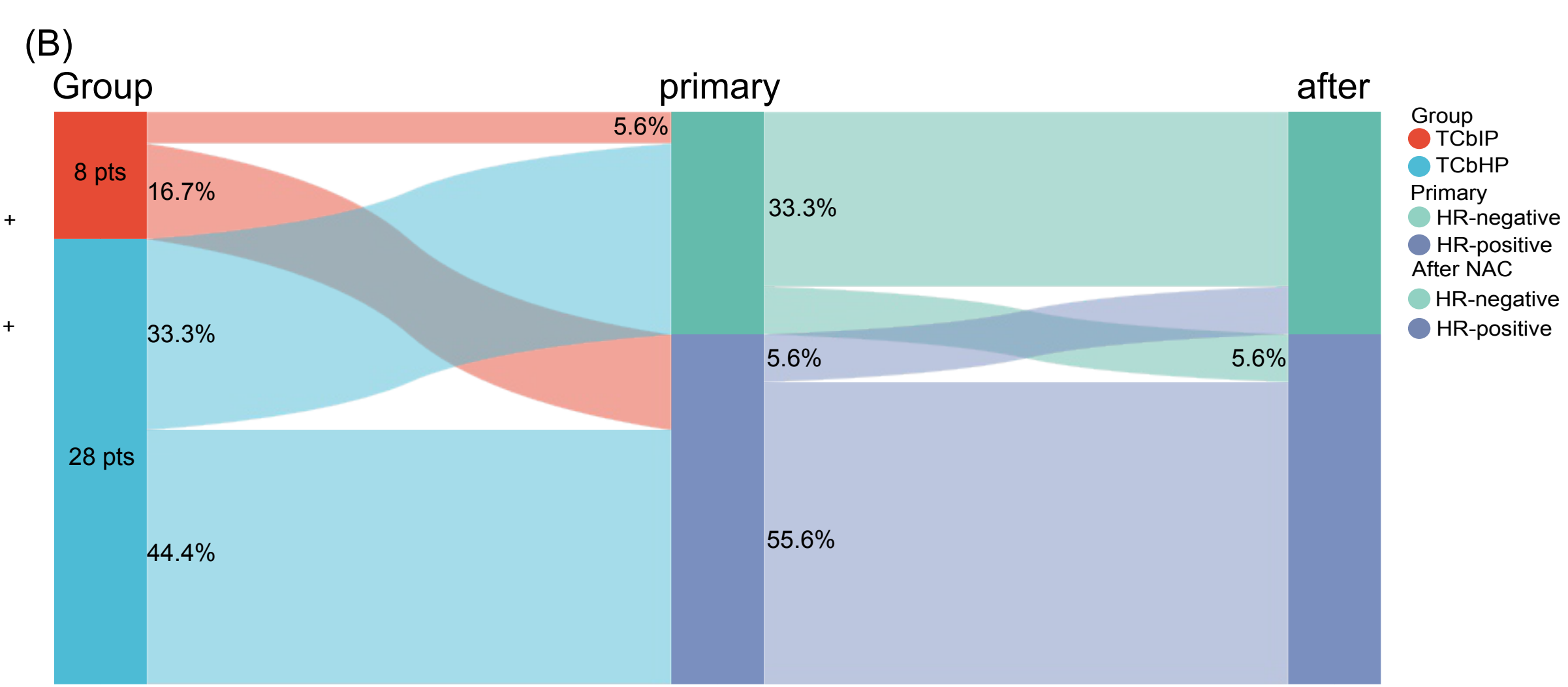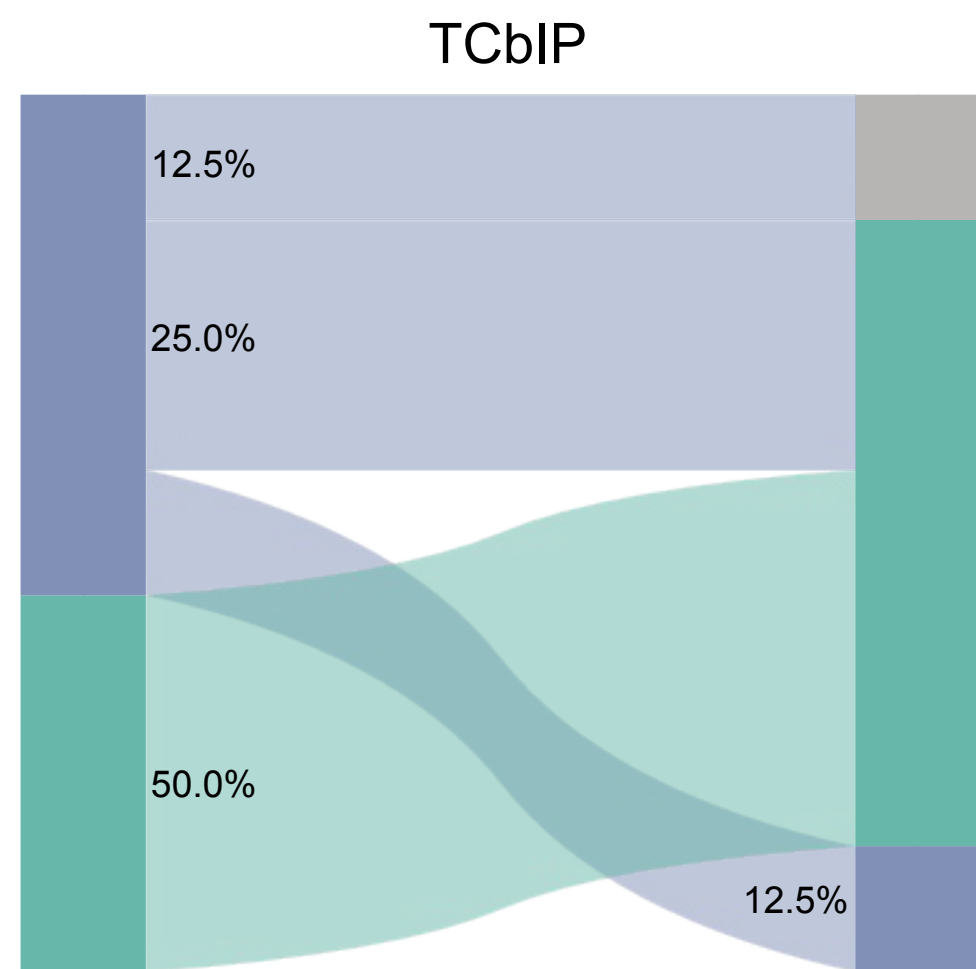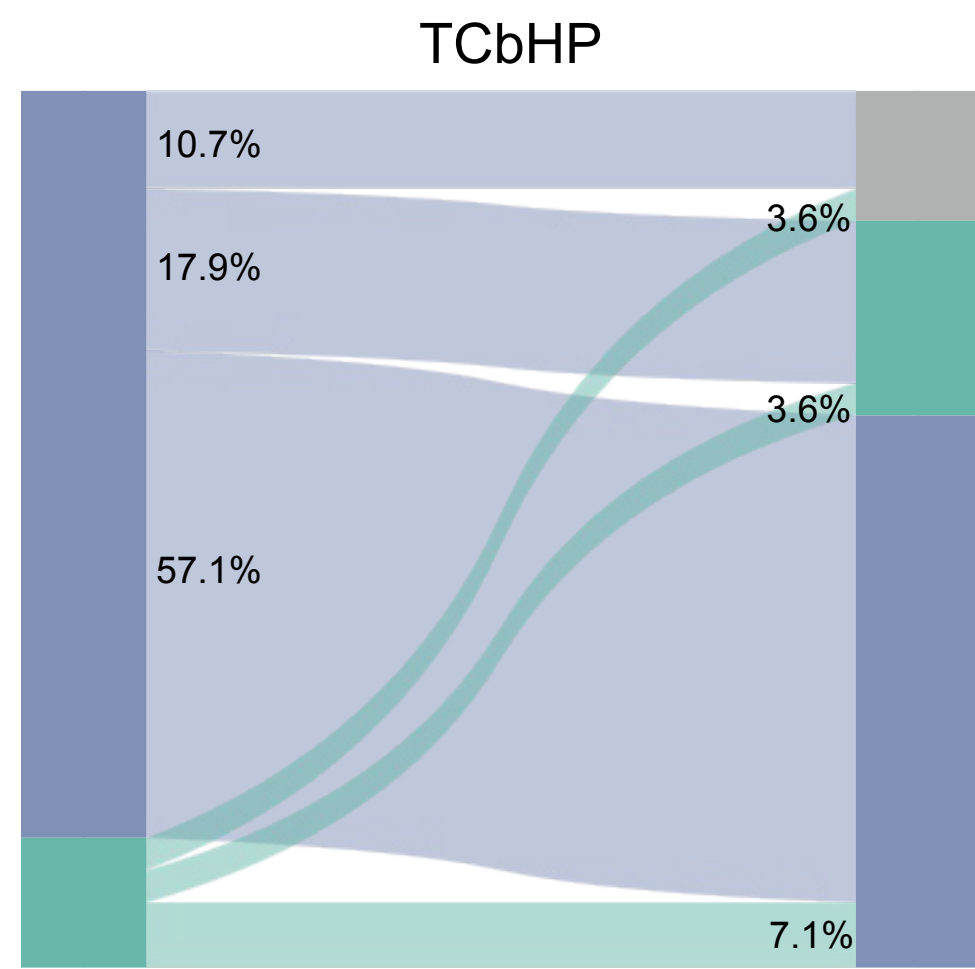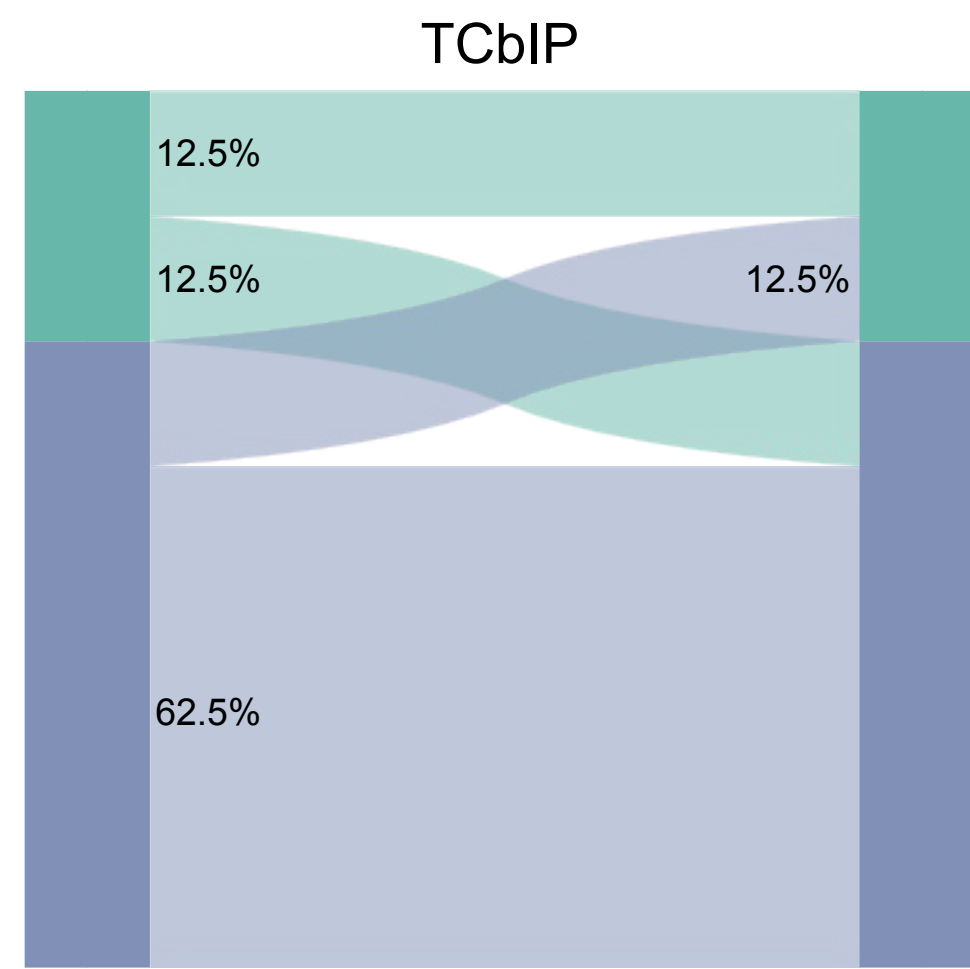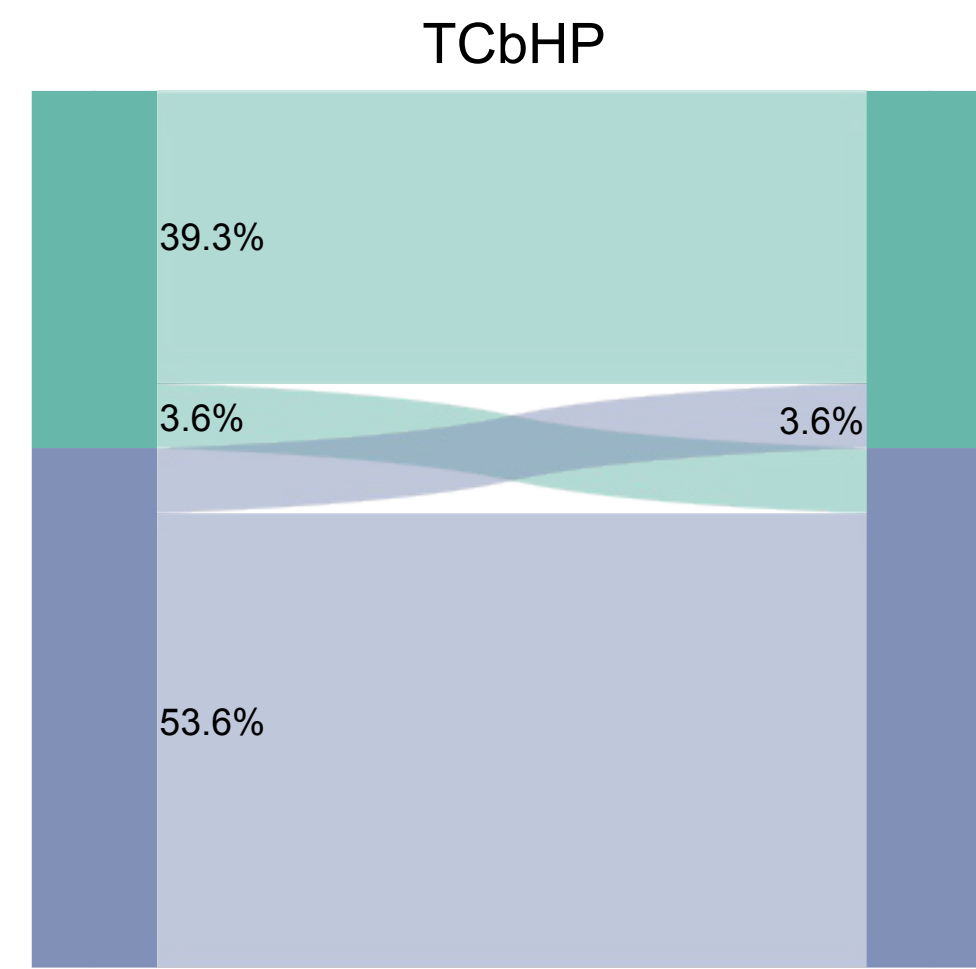

Supplement: Supplementary file 5 — Supplementary Material 5: Figure S3. Changes in HER2 expression and HR status between TCbIP and TCbHP group, following neoadjuvant treatment. (A) The Sankey diagrams show the changes in two groups with HER2 expression. (B) The Sankey diagrams show the changes in two groups with HR status. [file 12885_2024_12654_MOESM5_ESM.pdf]
